# Characteristics of Mycoplasma genitalium Urogenital Infections in a Diverse Patient Sample from the United States: Results from the Aptima Mycoplasma genitalium Evaluation Study (AMES)

**DOI:** 10.1128/JCM.00165-20

**Published:** 2020-06-24

**Authors:** Lisa E. Manhart, Charlotte A. Gaydos, Stephanie N. Taylor, Rebecca A. Lillis, Edward W. Hook, Jeffrey D. Klausner, Carmelle V. Remillard, Melissa Love, Byron McKinney, Damon K. Getman

**Affiliations:** a Department of Epidemiology, University of Washington, Seattle, Washington, USA; b Department of Global Health, University of Washington, Seattle, Washington, USA; c Center for AIDS and STD, University of Washington, Seattle, Washington, USA; d Division of Infectious Diseases, Johns Hopkins University, Baltimore, Maryland, USA; e Louisiana State University Health Sciences Center, New Orleans, Louisiana, USA; f Department of Medicine, University of Alabama, Birmingham, Birmingham, Alabama, USA; g Department of Epidemiology, University of Alabama, Birmingham, Birmingham, Alabama, USA; h Department of Microbiology, University of Alabama, Birmingham, Birmingham, Alabama, USA; i UCLA Division of Infectious Diseases, Department of Medicine, David Geffen School of Medicine, University of California, Los Angeles, California, USA; j Hologic, Inc., San Diego, California, USA; Marquette University

**Keywords:** Aptima, Aptima *Mycoplasma genitalium* Evaluation Study (AMES), *Mycoplasma genitalium*, epidemiology, sexually transmitted infection

## Abstract

Data from a large prospective multicenter clinical validation study of a nucleic acid amplification *in vitro* diagnostic test for Mycoplasma genitalium were analyzed to describe the prevalence of M. genitalium infection, risk factors, and disease associations in female and male patients seeking care in diverse geographic regions of the United States. Among 1,737 female and 1,563 male participants, the overall prevalence of M. genitalium infection was 10.3% and was significantly higher in persons ages 15 to 24 years than in persons ages 35 to 39 years (for females, 19.8% versus 4.7% [odds ratio {OR} = 5.05; 95% confidence interval {CI} = 3.01 to 8.46]; for males, 16.5% versus 9.4% [OR = 1.91; 95% CI = 1.20 to 3.02]). The risk for M. genitalium infection was higher in black than in white participants (for females, 12.0% versus 6.8% [OR = 1.88; 95% CI = 1.30 to 2.72]; for males, 12.9% versus 6.9% [OR = 2.02; 95% CI = 1.38 to 2.96]) and higher in non-Hispanic than in Hispanic participants (for females, 11.2% versus 6.0% [OR = 1.97; 95% CI = 1.25 to 3.10]; for males, 11.6% versus 6.8% [OR = 1.80; 95% CI = 1.14 to 2.85]). Participants reporting urogenital symptoms had a significantly elevated risk of M. genitalium infection compared to that for asymptomatic individuals (for females, OR = 1.53 [95% CI = 1.09 to 2.14]; for males, OR = 1.42 [95% CI = 1.02 to 1.99]). Women diagnosed with vaginitis and cervicitis had a higher prevalence of M. genitalium infection than women without those diagnoses, although this was statistically significant only for vaginitis (for vaginitis, OR = 1.88 [95% CI = 1.37 to 2.58]; for cervicitis, OR = 1.42 [95% CI = 0.61 to 2.96]). A diagnosis of urethritis in men was also significantly associated with M. genitalium infection (OR = 2.97; 95% CI = 2.14 to 4.13). Few characteristics distinguished asymptomatic from symptomatic M. genitalium infections. These results from persons seeking care in the United States suggest that M. genitalium infection should be considered in young persons presenting with urogenital symptoms.

## Abstract (precis)

Data from a large prospective multicenter clinical validation study of a nucleic acid amplification *in vitro* diagnostic test for Mycoplasma genitalium were analyzed to describe the prevalence of M. genitalium infection, risk factors, and disease associations in female and male patients seeking care in diverse geographic regions of the United States. Among 1,737 female and 1,563 male participants, the overall prevalence of M. genitalium infection was 10.

## INTRODUCTION

Reproductive tract disease syndromes account for substantial health care utilization. Approximately 60% of reproductive-age women have gynecologic or obstetric visits each year (1), and the last report of the number of physician office visits for male urethritis was approximately 200,000 annually (2). While sexually transmitted pathogens are not implicated in all of these situations, testing for them is often undertaken as part of the diagnostic assessment. Infection with Neisseria gonorrhoeae and Chlamydia trachomatis in women can result in pelvic inflammatory disease (PID) and serious sequelae, including ectopic pregnancy, infertility, and chronic pelvic pain (3). For these reasons, annual screening of women under age 25 years for these pathogens is recommended as a preventive measure (4). Infection with Mycoplasma genitalium has also been linked with female cervicitis, PID, and preterm delivery (5, 6), as well as with male urethritis (7), but the causal relationship between infection and adverse sequelae in women is not well understood, and screening is not currently recommended, as some of these associations may be inconsistent (6).

Despite the epidemiologic data indicating an association of M. genitalium infection with reproductive tract disease syndromes, incorporating M. genitalium diagnostics into initial clinical assessments of these syndromes has only recently become practical. While a number of nucleic acid amplification tests (NAAT), including research-use-only assays and Conformité Européenne (CE)-marked assays, have been in use since the early 1990s, the U.S. Food and Drug Administration (FDA) cleared the first M. genitalium NAAT, the Aptima Mycoplasma genitalium (AMG) assay (Hologic, Inc., San Diego, CA), in early 2019, paving the way for the more widespread consideration of this organism in clinical care in the United States. This is important, because recommended empirical therapy for these syndromes (6) is suboptimal for M. genitalium, and more widely available diagnostic tests will permit clinicians to better target appropriate treatment to the infecting pathogen.

To inform the use of M. genitalium NAATs in patient management, we analyzed data from the Aptima Mycoplasma genitalium Evaluation Study (AMES), a large prospective multicenter clinical study conducted to evaluate the assay (8). We estimated the prevalence of M. genitalium infections and evaluated the association of M. genitalium infection with reproductive tract symptoms, signs, and diagnoses in persons seeking care at geographically diverse locations in the United States.

## MATERIALS AND METHODS

### Study population and sample collection.

Persons with or without genitourinary sexually transmitted infection (STI) symptoms seen at participating sites were enrolled between July 2017 and April 2018 at 21 U.S. sites located in 6 regions of the country (8). Sexually active men and women at least 14 years of age were eligible. Persons were excluded if they had enrolled previously or had received antibiotics potentially active against M. genitalium (macrolides, fluoroquinolones, tetracyclines, or clindamycin) within 21 days of enrollment, based on the estimated time to clearance (9). Clinicians collected urogenital swab specimens during the routine clinic exam from eligible persons who provided consent and recorded the reported symptoms, clinical observations, and clinical diagnoses.

All participants provided first-void urine and swab specimens with a standardized order of collection. In men, the order was a clinician-collected urethral swab, a self-collected penile-meatal swab, and self-collected urine. In women, self-collected specimens were collected first (urine followed by a vaginal swab). During a speculum exam, clinicians then collected vaginal and endocervical swab specimens, in that order. All specimens were placed into Aptima tubes containing specimen transport medium and stored fresh (2°C to 30°C) or frozen (at –20°C or less) after collection. Specimens were tested first at regional laboratories with the investigational AMG assay on the Panther system, which detects M. genitalium 16S rRNA, and subsequently frozen and transported to Hologic, Inc., on dry ice for reference testing. Assay controls, including the strains used, have been previously described in detail (10).

M. genitalium infection was defined using a patient infected status (PIS) as previously described (8). This was comprised of results from the urethral swab specimens for men and patient-collected vaginal swab specimens for women tested with three validated research-use-only alternate transcription-mediated amplification (Alt TMA) assays developed by Hologic, Inc. Alt TMA assays targeted unique regions of M. genitalium 16S or 23S rRNA (8, 10). If at least two of three Alt TMA assay results were positive, the PIS was considered M. genitalium positive; if two Alt TMA assay results were negative, the PIS was considered M. genitalium negative. In validation studies, assay sensitivity was not affected by the freeze-thaw cycle prior to Alt TMA testing.

Participants were classified as symptomatic if they reported at least one of the following STI symptoms: an abnormal genital discharge, genital itching, pain/discomfort during sexual intercourse or during urination, or pain/discomfort in the groin or lower belly. Among asymptomatic persons, the reason for the clinic visit was documented. Clinical diagnoses were made according to the clinic’s standard of care.

### Statistical methods.

We estimated the prevalence and calculated the odds ratios (ORs) and 95% score confidence intervals (CIs) (11). Prevalence was tabulated by age, sex, symptom status, race/ethnicity, geographic area, and clinic type. Univariable odds ratios for the association of characteristics with M. genitalium infection were calculated separately by sex. Participants with an unknown PIS due to inconclusive results from reference testing with Alt TMA assays (*n* = 61) and/or samples with invalid or missing investigational assay results (*n* = 82) were excluded from the analyses. We performed multivariable logistic regression and evaluated potential confounding characteristics (age, race, other diagnoses), retaining those that had an appreciable influence on estimates of the relationship between M. genitalium infection and specific clinical diagnoses. Analyses were performed with SAS software (version 9.4; SAS Institute Inc., Cary, NC).

### Ethics approval.

Institutional review board approvals were obtained locally by all clinical centers. The study was conducted in accordance with the ethical principles derived from the Declaration of Helsinki and Belmont Report (12) and in compliance with the FDA and good clinical practice guidelines set forth by the International Conference on Harmonisation (ICH-E6) (13).

## RESULTS

### Characteristics of study population.

Of the 3,393 persons enrolled, 3,300 (97.3%) nonwithdrawn persons who provided specimens were evaluable and included in the analyses of assay performance (8). Of these, 1,737 were female and 1,563 were male (Table 1). Most women (61.0%) were black, with 34.0% being white and 1.7% being Asian. Among the men, the race/ethnicity distribution was similar to that for the women. Hispanic ethnicity was reported by 21.9% and 21.7% of the women and men, respectively. The women ranged in age from 15 to 74 years (median age, 29 years; interquartile range [IQR], 24 to 37 years). The age range for men was similar (16 to 82 years), but the median age was somewhat higher (median age, 33 years; IQR, 26 to 45 years). Overall, 43.2% of the participants were from the southeastern United States and 31.5% were from the southwestern United States. Sites in the Mid-Atlantic, Midwest, Northeast, and Northwest each contributed between 2.0% and 8.7% of participants. The majority of participants attended clinical research centers (39.2%), high-risk STI clinics (31.4%), or family planning clinics (18.5%). Emergency medicine clinics (3.1%), family medicine/obstetrics-gynecology (OB-GYN) clinics (0.6%), and non-STI public health clinics (7.1%) accounted for a lower proportion of all enrollees.

**TABLE 1:** Prevalence of M. genitalium urogenital infection by sociodemographic characteristic, geographic region, and enrollment clinic type^*a*^

Characteristic	M. genitalium infection prevalence							
	Female (*n* = 1,737)				Male (*n* = 1,563)			
	*n*/*N*	%	95% CI	OR (95% CI)	*n*/*N*	%	95% CI	OR (95% CI)
Age (yr)								
15–24	88/444	19.8	16.4, 23.8	5.05 (3.01, 8.46)	47/285	16.5	12.6, 21.2	1.91 (1.20, 3.02)
25–34	68/751	9.1	7.2, 11.3	2.03 (1.20, 3.43)	74/580	12.8	10.3, 15.7	1.41 (0.93, 2.14)
35–49	19/407	4.7	3.0, 7.2	Reference	37/394	9.4	6.9, 12.7	Reference
≥50	1/135	0.7	0.1, 4.1	0.15 (0.00, 0.98)	7/304	2.3	1.1, 4.7	0.23 (0.08, 0.53)
Race^*b*^								
White	40/591	6.8	5.0, 9.1	Reference	37/540	6.9	5.0, 9.3	Reference
Black	127/1,059	12.0	10.2, 14.1	1.88 (1.30, 2.72)	125/966	12.9	11.0, 15.2	2.02 (1.38, 2.96)
Asian	5/29	17.2	7.6, 34.5	2.87 (0.81, 8.22)	0/18	0.0	0.0, 17.6	NC
Unknown/other race	6/79	7.6	3.5, 15.6	1.13 (0.38, 2.82)	6/67	9.0	4.2, 18.2	1.34 (0.44, 3.37)
Ethnicity^*c*^								
Hispanic	23/381	6.0	4.1, 8.9	Reference	23/339	6.8	4.6, 10.0	Reference
Non-Hispanic	151/1,347	11.2	9.6, 13.0	1.97 (1.25, 3.10)	140/1,209	11.6	9.9, 13.5	1.80 (1.14, 2.85)
Collection site (region)^*d*^								
Mid-Atlantic	16/142	11.3	7.1, 17.5	Reference	13/118	11.0	6.6, 17.9	Reference
Midwest	23/190	12.1	8.2, 17.5	1.08 (0.55, 2.14)	14/98	14.3	8.7, 22.6	1.35 (0.60, 3.02)
Northeast	13/106	12.3	7.3, 19.9	1.10 (0.50, 2.40)	11/119	9.2	5.2, 15.8	0.82 (0.35, 1.92)
Northwest	0/12	0.0	0.0, 24.2	NC	3/53	5.7	1.9, 15.4	0.48 (0.09, 1.88)
Southeast	72/703	10.2	8.2, 12.7	0.90 (0.51, 1.60)	84/721	11.7	9.5, 14.2	1.07 (0.57, 1.98)
Southwest	52/584	8.9	6.9, 11.5	0.77 (0.43, 1.39)	40/454	8.8	6.5, 11.8	0.78 (0.40, 1.51)
Collection site (type)								
Clinical research center	43/625	6.9	5.1, 9.1	Reference	43/671	6.4	4.8, 8.5	Reference
Emergency medicine clinic	8/48	16.7	8.7, 29.6	2.71 (1.03, 6.34)	7/53	13.2	6.5, 24.8	2.22 (0.80, 5.36)
Family medicine/OB-GYN clinic	1/21	4.8	0.8, 22.7	0.68 (0.02, 4.44)				NC
Family planning clinic	39/378	10.3	7.6, 13.8	1.56 (0.99, 2.45)	28/232	12.1	8.5, 16.9	2.00 (1.21, 3.31)
Hospital system high-risk STI clinic	78/600	13.0	10.5, 15.9	2.02 (1.37, 2.99)	73/437	16.7	13.5, 20.5	2.93 (1.97, 4.36)
Public health clinic	7/65	10.8	5.3, 20.6	1.63 (0.59, 3.89)	14/170	8.2	5.0, 13.3	1.31 (0.70, 2.46)

a NC, not calculable; *n*/*N*, number of participants with urogenital M. genitalium infection/total number of participants with the indicated characteristic.

b Participants could report multiple responses.

c Ethnicity was self-reported as unknown by 9 female and 5 male participants.

d Mid-Atlantic: Maryland, North Carolina, and Washington, DC. Midwest: Indiana, Michigan, Nebraska, and Ohio (2 sites). Northeast: Connecticut and New Jersey. Northwest: Washington. Southeast: Alabama, Georgia, Florida (3 sites), and Louisiana. Southwest: California (2 sites) and Texas (2 sites).

As previously reported (8), the overall prevalence of M. genitalium infection was 10.3%. Prevalence was roughly similar in men and women: 10.1% in women and 10.6% in men.

### Association with sociodemographic characteristics.

The prevalence of M. genitalium infection was the highest in persons 15 to 24 years of age (19.8% in women, 16.5% in men; Table 1) and the lowest in persons ≥50 years of age (0.7% in women, 2.3% in men). Women ages 15 to 24 years were 5-fold more likely to have M. genitalium infection than women ages 35 to 49 years (OR = 5.05; 95% CI = 3.01 to 8.46), and men ages 15 to 24 years were approximately 2-fold more likely to have M. genitalium infection than men ages 35 to 49 years (OR = 1.91; 95% CI = 1.20 to 3.02). The prevalence of M. genitalium infection was similar in black women and men (12.0% and 12.9%, respectively), and black women and men were approximately twice as likely to have M. genitalium infection as white participants (for women, OR = 1.88 [95% CI = 1.30 to 2.72]; for men, OR = 2.02 [95% CI = 1.38 to 2.96]). In contrast, the prevalence of M. genitalium infection was lower in Hispanic persons (6.0% and 6.8% in women and men, respectively), and non-Hispanic men and women were approximately twice as likely to have M. genitalium infection as Hispanic persons (for women, OR = 1.97 [95% CI = 1.25 to 3.10]; for men, OR = 1.80 [95% CI = 1.14 to 2.85]).

The prevalence of M. genitalium infection among women was the lowest among those attending family medicine/OB-GYN clinics (4.8%) and clinical research centers (6.9%). It ranged from 10% to 13% in family planning clinic, STI clinic, and public health clinic attendees and was the highest in women seeking care in emergency medicine settings (16.7%). Women in those settings were nearly 3-fold more likely to have M. genitalium infection than women attending clinical research centers (OR = 2.71; 95% CI = 1.03 to 6.34). In contrast, among men, the prevalence of M. genitalium infection was the highest in STI clinic settings (16.7%). Men attending family planning clinics were twice as likely (OR = 2.00; 95% CI = 1.21 to 3.31) to have an M. genitalium infection as men attending clinical research centers, and those attending STI clinics were three times as likely (OR = 2.93; 95% CI = 1.97 to 4.36) to have an M. genitalium infection as men attending clinical research centers.

### Association with patient-reported symptoms.

Symptoms were reported by 61% of women and 55% of men (Table 2). The prevalence of M. genitalium infection in symptomatic women and men was similar (11.6% and 12.0%, respectively), and symptomatic persons were more likely to have M. genitalium infection than asymptomatic persons (for women, OR = 1.53 [95% CI = 1.09 to 2.14]; for men, OR = 1.42 [95% CI = 1.02 to 1.99]). Among women, prevalence was the highest among those reporting an abnormal vaginal odor (14.6%), pain during urination (14.4%), or an abnormal vaginal discharge (13.0%) and the lowest among those reporting abnormal vaginal bleeding (6.3%). Women who reported an abnormal vaginal odor (OR = 1.82; 95% CI = 1.31 to 2.52) and an abnormal vaginal discharge (OR = 1.67; 95% CI = 1.22 to 2.28) were significantly more likely than women who did not report each symptom to have M. genitalium infection. Among men, prevalence was the highest among those reporting penile or urethral discharge (20.4%) and the lowest among those reporting itching or tingling of the penis (7.4%). Penile or urethral discharge was the only symptom significantly associated with M. genitalium infection among men (OR = 2.77; 95% CI = 1.94 to 3.94).

**TABLE 2:** Prevalence of urogenital M. genitalium infection in participants reporting symptoms of urogenital sexually transmitted infection and association with symptoms

Patient-reported urogenital symptoms^*a*^	M. genitalium infection prevalence			
	Female (*n* = 1,737)		Male (*n* = 1,563)	
	*n*/*N*^*b*^ (%)	OR^*c*^ (95% CI)	*n*/*N* (%)	OR^*c*^ (95% CI)
Any reported symptom	122/1,053 (11.6)	1.53 (1.09, 2.14)	104/866 (12.0)	1.42 (1.02, 1.99)
Pain/discomfort in groin or lower belly	17/159 (10.7)	1.07 (0.63, 1.81)	12/149 (8.1)	0.72 (0.39, 1.33)
Pain/burning/discomfort during urination	18/125 (14.4)	1.55 (0.92, 2.62)	39/358 (10.9)	1.05 (0.72, 1.53)
Pain/discomfort during sexual intercourse	11/106 (10.4)	1.03 (0.54, 1.96)	8/65 (12.3)	1.20 (0.48, 2.59)
Genital blisters/sores/bumps/rash/warts	7/69 (10.1)	1.00 (0.38, 2.24)	9/94 (9.6)	0.89 (0.39, 1.82)
Abnormal vaginal odor	65/445 (14.6)	1.82 (1.31, 2.52)		
Vaginal/vulvar itching or irritation	51/429 (11.9)	1.28 (0.90, 1.80)		
Abnormal vaginal bleeding	4/63 (6.3)	0.59 (0.15, 1.63)		
Abnormal vaginal discharge	90/692 (13.0)	1.67 (1.22, 2.28)		
Penile/urethral discharge			56/275 (20.4)	2.77 (1.94, 3.94)
Burning/itching around opening of penis			22/269 (8.2)	0.72 (0.45, 1.15)
Itching/tingling on the inside of penis			13/175 (7.4)	0.65 (0.36, 1.18)

a Participants could report multiple symptoms.

b *n*/*N*, number of participants with urogenital M. genitalium infection/total number of participants with the indicated symptoms.

c The referent category in all cases is the absence of the symptom.

### Association with clinical signs and diagnoses.

Relatively few clinical signs noted during examination were associated with M. genitalium infection (Table 3). Among women, only clinician-observed blisters/sores/bumps/rash/warts in the genital region (OR = 3.39; 95% CI = 1.72 to 6.69) and a clinician-observed abnormal vaginal odor (OR = 1.65; 95% CI = 1.16 to 2.33) were associated with M. genitalium infection. Despite a nonsignificant association between M. genitalium infection and an abnormal vaginal discharge (OR = 1.32; 95% CI = 0.97 to 1.81), diagnoses of vaginitis were significantly more common among M. genitalium-infected women (OR = 1.88; 95% CI = 1.37 to 2.58) than among those not diagnosed with vaginitis. Although the risk of a cervicitis diagnosis was somewhat elevated among women with M. genitalium infection, this was not statistically significant (OR = 1.42; 95% CI = 0.61 to 2.96). Observations of lower abdominal and/or pelvic tenderness in women were infrequent and not associated with M. genitalium infection. Diagnoses of pelvic inflammatory disease (PID) were even more infrequent, occurring in only 11 women (0.6%). M. genitalium infection was detected in 2 of 11 women with PID diagnoses, but the relationship between M. genitalium infection and PID was not statistically significant. Among men, both clinical signs of swollen lymph nodes in the groin (OR = 2.87; 95% CI = 1.42 to 5.77) and an abnormal urethral discharge (OR = 2.34; 95% CI = 1.61 to 3.40) were significantly associated with M. genitalium infection. Consistent with this was the significantly increased risk of a urethritis diagnosis among men with M. genitalium infection (OR = 2.97; 95% CI = 2.14 to 4.13). No other clinical diagnoses were significantly associated with M. genitalium infection in men.

**TABLE 3:** Prevalence of clinical findings and association of clinical findings with urogenital M. genitalium infection^*a*^

Clinical finding	M. genitalium infection prevalence			
	Female (*n* = 1,737)		Male (*n* = 1,563)	
	*n*/*N* (%)	OR^*b*^ (95% CI)	*n*/*N* (%)	OR^*b*^ (95% CI)
Clinician-reported urogenital signs^*c*^				
Any sign of urogenital infection	115/1,034 (11.1)	1.32 (0.95, 1.83)	77/608 (12.7)	1.43 (1.03, 1.98)
Swollen lymph nodes in groin	0	NC	11/45 (24.4)	2.87 (1.42, 5.77)
Genital blisters/sores/bumps/rash/warts	12/45 (26.7)	3.39 (1.72, 6.69)	11/135 (8.1)	0.73 (0.39, 1.39)
Abnormal vaginal odor	51/361 (14.1)	1.65 (1.16, 2.33)		
Abnormal vaginal discharge	98/858 (11.4)	1.32 (0.97, 1.81)		
Clear	6/58 (10.3)	Reference		
White	58/536 (10.8)	1.05 (0.43, 3.13)^*d*^		
Pink, bloody, brown, gray, other	18/156 (11.5)	1.13 (0.40, 3.67)^*d*^		
Yellow, green (pus-like)	16/108 (14.8)	1.51 (0.52, 4.99)^*d*^		
Urethral erythema	0	NC	17/207 (8.2)	0.73 (0.43, 1.23)
Abnormal urethral discharge	1/16 (6.3)	0.59 (0.01, 3.87)	46/244 (18.9)	2.34 (1.61, 3.40)
Lower abdominal/pelvic tenderness	1/35 (2.9)	0.26 (0.01, 1.55)	1/5 (20.0)	2.13 (0.04, 21.63)
Pain or swelling of testicles			3/25 (12.0)	1.16 (0.22, 3.92)
Clinician's diagnosis^*c*^				
Any clinical finding	122/1,024 (11.9)	1.65 (1.18, 2.31)	103/755 (13.6)	1.90 (1.36, 2.65)
Cervicitis	9/66 (13.6)	1.42 (0.61, 2.96)		
Pelvic inflammatory disease	2/11 (18.2)	1.98 (0.21, 9.68)		
Vaginitis	101/752 (13.4)	1.88 (1.37, 2.58)		
Cystitis	1/13 (7.7)	0.74 (0.02, 5.04)	1/9 (11.1)	1.06 (0.02, 7.99)
Urethritis	0/2 (0.0)	NC	83/438 (18.9)	2.97 (2.14, 4.13)
Abdominal/pelvic pain	2/17 (11.8)	1.18 (0.13, 5.16)	0	NC
Genital lesions	0/4 (0.0)	NC	1/12 (8.3)	0.77 (0.02, 5.35)
Genital warts	1/2 (50.0)	8.91 (0.11, 700.16)	4/31 (12.9)	1.26 (0.32, 3.69)
Urinary tract infection	0/16 (0.0)	NC	0	NC
HSV	3/13 (23.1)	2.69 (0.47, 10.57)	0/9 (0.0)	NC
Other,^*e*^ not available, unknown	13/186 (7.0)	0.64 (0.36, 1.15)	15/264(5.7)	0.46 (0.27, 0.80)

a HSV, herpes simplex virus infection; *n*/*N*, number of patients with urogenital M. genitalium infection/total number of patients with the indicated clinical finding; NC, not calculable.

b Unless otherwise noted, the referent category is the absence of the sign or diagnosis.

c The clinician could report multiple signs or diagnoses.

d The referent is clear abnormal vaginal discharge.

e Includes balanitis, proctitis, and lymphadenopathy.

In multivariable analyses, the relationships between M. genitalium infection and clinical diagnoses were somewhat attenuated after adjusting for age and race. Among women, the association between M. genitalium infection and vaginitis remained statistically significant (adjusted OR [AOR] = 1.54; 95% CI = 1.13 to 2.14). However, the relationship between M. genitalium infection and cervicitis (AOR = 1.08; 95% CI = 0.51 to 2.26) was no longer present in the adjusted analyses. Due to the small number of women with PID, estimates were unstable, and the results of multivariable analyses are not presented. Among men, the relationship between M. genitalium infection and urethritis remained statistically significant (AOR = 2.50; 95% CI = 1.77 to 3.53).

### Characteristics associated with asymptomatic infection.

Overall, 39% of women and 45% of men were asymptomatic (Table 4). The prevalence of M. genitalium infection was higher in symptomatic women and men than in asymptomatic women and men in almost all subgroups of the population, consistent with the observed association between M. genitalium infection and urogenital symptoms (Table 2).

**TABLE 4:** Characteristics associated with asymptomatic urogenital M. genitalium infection^*a*^

Characteristic	M. genitalium infection prevalence					
	Females			Males		
	% (*n*/*N*)		OR^*c*^ (95% CI)	% (*n*/*N*)		OR^*c*^ (95% CI)
	Sym^*b*^ (*n* = 1,053)	ASym^*b*^ (*n* = 684)		Sym^*b*^ (*n* = 866)	ASym^*b*^ (*n* = 697)	
Age (yr)						
15–24	21.4 (65/304)	16.4 (23/140)	1.38 (0.82, 2.34)	18.8 (30/160)	13.6 (17/125)	1.47 (0.77, 2.80)
25–34	10.2 (46/452)	7.4 (22/299)	1.43 (0.84, 2.42)	14.9 (46/309)	10.3 (28/271)	1.52 (0.92, 2.51)
35–49	4.0 (10/247)	5.6 (9/160)	0.71 (0.25, 2.02)	11.7 (25/213)	6.6 (12/181)	1.87 (0.91, 3.84)
≥50	2.0 (1/50)	0.0 (0/85)	Inf (0.09, Inf)	1.6 (3/184)	3.3 (4/120)	0.48 (0.07, 2.90)
Race^*d*^						
White	7.4 (23/310)	6.0 (17/281)	1.24 (0.65, 2.38)	8.5 (22/259)	5.3 (15/281)	1.65 (0.83, 3.25)
Black	13.6 (92/677)	9.2 (35/382)	1.56 (1.03, 2.35)	13.9 (81/584)	11.5 (44/382)	1.24 (0.84, 1.83)
Asian	23.8 (5/21)	0.0 (0/8)	Inf (0.49, Inf)	0.0 (0/9)	0.0 (0/9)	NC
Unknown/other race	7.0 (4/57)	9.1 (2/22)	0.75 (0.10, 8.99)	11.5 (3/26)	7.3 (3/41)	1.65 (0.20, 13.30)
Ethnicity^*e*^						
Hispanic	6.7 (14/210)	5.3 (9/171)	1.29 (0.50, 3.46)	7.3 (12/164)	6.3 (11/175)	1.18 (0.50, 2.75)
Non-Hispanic	12.6 (106/838)	8.8 (45/509)	1.49 (1.03, 2.16)	13.0 (90/694)	9.7 (50/515)	1.39 (0.96, 2.00)
Collection site (region)^*f*^						
Mid-Atlantic	13.2 (9/68)	9.5 (7/74)	1.46 (0.45, 4.91)	11.7 (9/77)	9.8 (4/41)	1.22 (0.31, 5.81)
Midwest	12.1 (17/141)	12.2 (6/49)	0.98 (0.34, 3.24)	13.2 (7/53)	15.6 (7/45)	0.83 (0.23, 3.04)
Northeast	12.7 (9/71)	11.4 (4/35)	1.13 (0.29, 5.39)	12.9 (4/31)	8.0 (7/88)	1.71 (0.34, 7.34)
Northwest	0.0 (0/9)	0.0 (0/3)	NC	6.4 (3/47)	0.0 (0/6)	Inf (0.07, Inf)
Southeast	11.8 (54/459)	7.4 (18/244)	1.67 (0.96, 2.92)	12.1 (60/494)	10.6 (24/227)	1.17 (0.71, 1.93)
Southwest	10.8 (33/305)	6.8 (19/279)	1.66 (0.92, 2.99)	12.8 (21/164)	6.6 (19/290)	2.09 (1.09, 4.02)
Collection site (type)						
Clinical research center	6.6 (19/287)	7.1 (24/338)	0.93 (0.50, 1.73)	6.0 (23/383)	6.9 (20/288)	0.86 (0.46, 1.59)
Emergency medicine clinic	15.2 (7/46)	50.0 (1/2)	0.18 (0.00, 16.09)	15.2 (7/46)	0.0 (0/7)	Inf (0.27, Inf)
Family medicine/OB-GYN clinic	0.0 (0/5)	6.3 (1/16)	NC	NC	NC	NC
Family planning clinic	13.0 (31/238)	5.7 (8/140)	2.47 (1.07, 6.40)	16.3 (15/92)	9.3 (13/140)	1.90 (0.86, 4.21)
Hospital system high-risk STI clinic	13.7 (62/452)	10.8 (16/148)	1.31 (0.73, 2.35)	17.6 (51/289)	14.9 (22/148)	1.23 (0.71, 2.12)
Public health clinic	12.0 (3/25)	10.0 (4/40)	1.23 (0.16, 7.99)	14.3 (8/56)	5.3 (6/114)	3.00 (0.85, 11.03)

a Asym, asymptomatic; Inf, infinity; *n*/*N*, number of patients with urogenital M. genitalium infection/total number of patients with the indicated characteristic; NC, not calculable; Sym, symptomatic.

b Symptom status is determined based on patient-reported symptoms.

c Odds ratio represents the association of M. genitalium with symptoms in each subgroup. The referent category in all cases is asymptomatic participants.

d Participants could report multiple responses.

e Ethnicity was self-reported as unknown by 5 female and 8 male participants.

f Mid-Atlantic: Maryland, North Carolina, and Washington, DC. Midwest: Indiana, Michigan, Nebraska, and Ohio (2 sites). Northeast: Connecticut and New Jersey. Northwest: Washington. Southeast: Alabama, Georgia, Florida (3 sites), and Louisiana. Southwest: California (2 sites) and Texas (2 sites).

The relationship between M. genitalium infection and reported symptoms was statistically significant in only four groups. Symptomatic women who were black (OR = 1.56; 95% CI = 1.03 to 2.35), non-Hispanic (OR = 1.49; 95% CI = 1.03 to 2.16), or enrolled at family planning clinics (OR = 2.47; 95% CI = 1.07 to 6.40) were significantly more likely to have M. genitalium infection than asymptomatic women in those groups. Among men from the Southwest United States, the prevalence of M. genitalium infection was significantly higher (OR = 2.09; 95% CI = 1.09 to 4.02) in symptomatic men than in asymptomatic men. No other significant associations with symptom status were identified.

Among asymptomatic participants, the reason for the clinic visit was not specified in 15.6% of women and 33.7% of men (data not shown). In participants with a documented reason for the visit, the prevalence of M. genitalium infection was the highest among those seeking care because of known contact with a person with a confirmed or suspected STI (11.6% in women, 13.7% in men). Among women, women presenting for STI screening or for testing because of contact with a partner with an STI were 2- to 3-fold more likely to have M. genitalium infection than women presenting to the clinic for a routine pelvic exam, although the latter was not statistically significant (for screening, OR = 2.05 [95% CI = 1.07 to 3.93]; for contact, OR = 2.84 [95% CI = 0.87 to 9.24]).

## DISCUSSION

We estimated the prevalence of M. genitalium infection and disease associations in a large, diverse population of patients from broad geographic settings across the United States. Participants were enrolled in a prospective multicenter clinical performance evaluation study conducted to validate the AMG assay, an FDA-cleared (510k# DEN180047) *in vitro* diagnostic NAAT (8). The prevalence of urogenital M. genitalium infection was approximately 10%, slightly lower than previous reports of the prevalence in mostly symptomatic populations (14, 15), reflecting the mix of symptomatic and asymptomatic persons in our study. Prevalence was higher in younger persons and in those of black race or non-Hispanic ethnicity, as well as among women attending emergency medicine clinics. The prevalence of M. genitalium infection was also higher among symptomatic persons than among asymptomatic persons, with significant associations between M. genitalium infection and vaginitis in women and between M. genitalium infection and urethritis in men. Few characteristics differentiated symptomatic from asymptomatic M. genitalium infections. In asymptomatic study participants, the only reason for a clinic visit that was associated with M. genitalium infection was seeking care for screening, and this was true only for women.

The association between M. genitalium infection and young age is consistent with previous reports (16–18). Whereas prevalence was the highest in 15- to 24-year-olds overall, age-related prevalence dropped substantially in women ages 25 to 34 years (from nearly 20% to 9.1%), and it was only slightly lower in men ages 25 to 34 years (16.5% versus 12.8%). This is somewhat similar to results from the Natsal 3 study, which demonstrated a clear linear decrease in the prevalence of M. genitalium infection with age in women, but in men, the highest prevalence was in the 25- to 34-year-old age group (16). This may reflect typical sexual mixing patterns, where young women often have older male partners and therefore often have a higher prevalence of STIs than males of the same age (17).

The association of M. genitalium infection with vaginitis is of interest and is perhaps substantiated by the accompanying association with an abnormal vaginal odor. This symptom is typically associated with bacterial vaginosis (BV) (19), and vaginal symptoms have not been frequently associated with M. genitalium infection (17). Indeed, a recent study evaluating the syndromic management of vaginal discharge concluded that an abnormal vaginal discharge was not a sensitive criterion for capturing M. genitalium infection (20). Two other previous studies have reported an increased risk of acquiring M. genitalium among women with BV (21, 22), and the association with vaginitis observed here may reflect an association with BV, although studies are inconsistent. Trichomonas vaginalis, another known cause of vaginitis, has also been associated with M. genitalium infection (23–25). Regrettably, neither a BV diagnosis nor T. vaginalis test results were provided in the context of this study, so we were unable to evaluate the extent to which BV or T. vaginalis infection might explain this association. In our clinical performance study (8), the sensitivity of the investigational AMG assay was the highest with self-obtained vaginal swab samples (98.9%) and lower with endocervical swab samples (81.5%), and self-obtained vaginal swabs were the preferred sample type. This supports the possibility that M. genitalium causes vaginal as well as cervical infection, and this warrants further investigation.

There was no association between clinician-recorded diagnoses of cervicitis and M. genitalium infection after adjustment for race and age, consistent with the findings of other studies (26). However, we did not have access to medical records to corroborate these reported diagnoses with objective evidence of cervicitis (e.g., easily induced cervical bleeding, elevated polymorphonuclear leukocyte [PMN] counts). Given the decreased availability of microscopy in many clinics, PMNs are often not quantitated, potentially reducing the specificity of a cervicitis diagnosis. In previous studies, the association between M. genitalium infection and cervicitis has been the strongest in those that defined cervicitis as ≥30 PMNs/high-power field in cervical exudates (27).

PID was rare in this population; it was diagnosed in less than 1% of women, restricting our ability to assess its association with M. genitalium infection. This limitation is not unique to our study. The POPI trial, a large randomized trial of chlamydial screening, observed a similarly low rate of PID (1.6%) (28). Study populations with a higher incidence of PID that will provide greater statistical power will be needed to definitively determine the role of M. genitalium in PID. Although swollen inguinal lymph nodes were infrequently observed, the association between M. genitalium infection and swollen inguinal lymph nodes suggests that M. genitalium may cause syndromes other than urethritis in men. However, although M. genitalium has been detected in men with epididymitis and in men with proctitis, to date no studies have demonstrated statistically significant associations with these syndromes (29, 30).

While it was not surprising that the prevalence of M. genitalium infection was high in STI clinics, the highest prevalence of M. genitalium infection in women was observed in those attending emergency medicine clinics. In the United States, many symptomatic persons attend emergency medicine clinics because they do not have a regular health care provider, often because they lack health insurance. These persons may also be at higher risk of STIs. The relatively higher prevalence in family planning clinic attendees was also somewhat surprising but may reflect the increasing use of these clinics for a variety of sexual health care needs, including care for symptoms of reproductive tract syndromes and STI screening when STI clinics are not readily accessible or when these clinics are not the care location of choice. Providers in these clinics may need to have a higher index of suspicion for M. genitalium infection and may consider testing symptomatic women for M. genitalium infection as part of clinical management.

There are a number of strengths and limitations to this study. Strengths include the large sample size as well as the variety of geographic locations and clinic types included. The AMG assay is highly sensitive and specific, resulting in minimal misclassification of M. genitalium infection status (8, 31). The reliance on clinical diagnoses may have resulted in some misclassification of syndrome status, but this reflects the situation in many clinical settings; most do not have the capacity to perform microscopy, and speculum exams are becoming increasingly less common. We also did not have access to laboratory results for other common STIs that are associated with the clinical conditions that we evaluated (e.g., C. trachomatis, N. gonorrhoeae, and T. vaginalis infections and BV) and therefore could not adjust for these causes of the syndromes that we evaluated. Up to 20% of persons infected with M. genitalium are coinfected with another STI pathogen (15). We lacked information on the sex of the sex partners, HIV infection status, and high-risk behaviors and could not assess their relationship with M. genitalium infection. Although antibiotic resistance in M. genitalium is of substantial concern, with rates exceeding 60% in many regions of the United States (15), this study was not designed to evaluate this. Future surveillance studies of the prevalence and distribution of resistance will be important.

In summary, the prevalence of M. genitalium infection in this study population of high-risk individuals (e.g., individuals reporting symptoms consistent with an STI or known contact with person with a confirmed or suspected STI) and low-risk individuals (e.g., asymptomatic individuals undergoing routine pelvic examination) was high and associated with many of the same characteristics elucidated in previous reports. Women seeking care in emergency medicine clinics, women with vaginitis, and men with urethritis were most likely to have M. genitalium infection in this study. Clinicians encountering symptomatic patients in these settings or with these syndromes should consider M. genitalium as an etiology.
